# In-silico prediction of disorder content using hybrid sequence representation

**DOI:** 10.1186/1471-2105-12-245

**Published:** 2011-06-17

**Authors:** Marcin J Mizianty, Tuo Zhang, Bin Xue, Yaoqi Zhou, A Keith Dunker, Vladimir N Uversky, Lukasz Kurgan

**Affiliations:** 1 Department of Electrical and Computer Engineering, University of Alberta, Edmonton, Alberta T6G 2V4, Canada; 2 Institute for Intrinsically Disordered Protein Research, Center for Computational Biology and Bioinformatics, Indiana University Schools of Medicine and Informatics, Indianapolis, Indiana 46202, USA; 3 Indiana University School of Informatics, Indiana University-Purdue University, Indianapolis, Indiana 46202, USA; 4 Department of Molecular Medicine, University of South Florida, Tampa, Florida 33612, USA; 5 Institute for Biological Instrumentation, Russian Academy of Sciences, 142290 Pushchino, Moscow Region, Russia

## Abstract

**Background:**

Intrinsically disordered proteins play important roles in various cellular activities and their prevalence was implicated in a number of human diseases. The knowledge of the content of the intrinsic disorder in proteins is useful for a variety of studies including estimation of the abundance of disorder in protein families, classes, and complete proteomes, and for the analysis of disorder-related protein functions. The above investigations currently utilize the disorder content derived from the per-residue disorder predictions. We show that these predictions may over-or under-predict the overall amount of disorder, which motivates development of novel tools for direct and accurate sequence-based prediction of the disorder content.

**Results:**

We hypothesize that sequence-level aggregation of input information may provide more accurate content prediction when compared with the content extracted from the local window-based residue-level disorder predictors. We propose a novel predictor, DisCon, that takes advantage of a small set of 29 custom-designed descriptors that aggregate and hybridize information concerning sequence, evolutionary profiles, and predicted secondary structure, solvent accessibility, flexibility, and annotation of globular domains. Using these descriptors and a ridge regression model, DisCon predicts the content with low, 0.05, mean squared error and high, 0.68, Pearson correlation. This is a statistically significant improvement over the content computed from outputs of ten modern disorder predictors on a test dataset with proteins that share low sequence identity with the training sequences. The proposed predictive model is analyzed to discuss factors related to the prediction of the disorder content.

**Conclusions:**

DisCon is a high-quality alternative for high-throughput annotation of the disorder content. We also empirically demonstrate that the DisCon's predictions can be used to improve binary annotations of the disordered residues from the real-value disorder propensities generated by current residue-level disorder predictors. The web server that implements the DisCon is available at http://biomine.ece.ualberta.ca/DisCon/.

## Background

The intrinsically disordered proteins (IDPs), also referred to as natively unfolded or intrinsically unstructured proteins, lack stable tertiary structure *in vitro*. These proteins are implicated in numerous processes including cellular signal transduction, transcriptional regulation, and translation [1], and their prevalence was demonstrated in several human diseases [2,3], including cancer [4], cardiovascular disease [5], neurodegenerative diseases [6,7], genetic diseases [8], and amyloidoses [9]. At the same time, the annotations of the IDPs are accumulated at a relatively low pace when compared with the growth of the number of known, non-redundant protein sequences. Over the last decade numerous sequence-derived characteristics, including low complexity [10], which was proposed in [11], high net charge and low content of hydrophobic amino acids [12,13], lack of regular secondary structure [14], to name just a few, were found to differentiate between disordered and ordered regions. The abovementioned results suggest that disorder can be predicted from the sequence and they motivate the development of computational models for the prediction of the disordered regions. Several such predictors were already developed; see [15] for a recent review. Majority of the existing predictors generate the disorder predictions for each residue in the input protein chain. These per-residue predictors can be divided into 4 categories: i) methods that utilize the *relative propensity *of amino acids to form disorder/ordered regions which include GlobPlot [16], IUPred [17], FoldIndex [18], and Ucon [19]; ii) methods that are based on classifiers generated with the help of *machine learning *algorithms, such as DISpro [20], DISOPRED [21], DISOPRED2 [22], PrDOS [23], POODLE predictors [24,25], PONDR predictors [10,26,27], Spritz [28], PROFbval [29], DisPSSMP [30], DisPSSMP2 [31], IUP [32], NORSnet [33] and OnD-CRFs [34]; iii) *meta-approach *methods that are based on a consensus of multiple base predictors including MULTICOM (also called PreDisorder) [35,36], metaPrDOS [37] and recent MD [38], MFDp [39], and PONDR-FIT [40] predictors; and iv) approaches that find disordered residues through an analysis of the *predicted 3D structural *models such as PrDOS [23] and DISOclust [41]. There are also methods that predict the propensity of the entire protein chain to be unstructured [13,42-44]. One of these approaches is based on the charge-hydropathy plots [13] and another utilizes distributions of the predicted per-residue disorder scores [42-44]. The abovementioned per-residue and per-chain methods perform the predictions in a high-throughput manner and consequently they can be used as a possible solution to close the annotation gap.

Although the per-residue methods are successful in the disorder prediction at the residue level, i.e., they achieve AUC (area under the ROC curve) of about 0.8 [38-40] and MCC (Matthews Correlation Coefficient) of about 0.45 [39] when tested on large benchmark datasets, we observe that they typically make relatively substantial mistakes at the sequence-level. More specifically, these methods may over-or under-predict the overall amount of disorder in the sequence. Tests of 10 recent disorder predictors that include methods from all four groups on a benchmark dataset of 200 chains, see Table 1, show that the mean average (over the dataset) squared errors between the native and the predicted amount of disorder vary between 0.07 and 0.18 (see the Results and Discussion sections). One of the potential reasons for these errors is the fact that virtually all of the most accurate recent predictors, such as NORSnet, DISOPRED2, MD, PONDR-FIT, and MFDp, use a local sequence window to predict the disorder while the information encoded in the entire chain may reveal an overall sequence-level disorder bias. The disorder predictors use identical (for all chains) cut-off values to annotate disordered residues based on the predicted real-value propensities, and we show that these annotations can be improved if the cut-off is adjusted to match the native amount of the disorder in the entire chain, which suggests that the knowledge of this sequence bias could be useful.

**Table 1:** Comparison of predictive quality of the DisCon and the disorder content extracted from the predictions of the 10 considered modern disorder predictors on the test dataset.

Predictor	Evaluation of the predicted disorder content										Evaluation of the predicted disorder at the residue-level		
	
	MSE		MAE		PCC		% of chains		MAE				
				
	value	**stat. signif**.	value	**stat. signif**.	value	**stat. signif**.	over-predicted	under-predicted	over-predicted	under-predicted	AUC	Accuracy	MCC
PROFbval	0.178	++	0.387	++	0.38	++	0.86	0.14	0.41	0.27	0.696	0.528	0.196
NORSnet	0.112	++	0.206	++	0.34	++	0.22	0.74	0.23	0.21	0.711	0.763	0.269
DISOclust	0.103	++	0.256	++	0.54	++	0.84	0.16	0.26	0.24	0.778	0.672	0.351
IUPRedL	0.083	++	0.172	=	0.47	++	0.40	0.57	0.14	0.20	0.767	0.785	0.365
MD	0.079	++	0.182	+	0.61	++	0.54	0.44	0.24	0.12	**0.816**	0.790	0.424
DISOPRED 2	0.076	++	0.167	=	0.49	++	0.57	0.41	0.14	0.22	0.780	0.771	0.382
MFDp	0.074	++	0.177	=	0.58	++	0.67	0.30	0.18	0.19	0.795	0.764	**0.425**
IUPRedS	0.070	+	**0.155**	=	0.53	++	0.49	0.48	0.10	0.22	0.771	**0.795**	0.366
Ucon	0.069	+	0.177	=	0.52	++	0.63	0.35	0.14	0.26	0.732	0.739	0.284
PONDR-FIT	0.066	+	0.167	=	0.55	++	0.65	0.34	0.13	0.24	0.776	0.777	0.383
DisCon	**0.050**		0.156		**0.68**		0.62	0.37	0.14	0.18	N/A	N/A	N/A

We report the MSE, MAE, and PCC values, the percentage of chains that are over-predicted (predicted with content higher than the native content) and under-predicted, and the MAE value for the over-and under-predicted chains. The methods are sorted in the descending order by the MSE values and the best values are shown in bold font. Results of the tests of significance of the differences between DisCon and the other methods are given in the "stat. signif." columns. The tests compare the absolute and the squared errors per-chain over all 200 chains in our test dataset, and Pearson correlation computed for 200 randomly selected sets of 100 proteins from the test dataset. The ++ and + denote that DisCon is statistically significantly better with *p *< 0.01 and *p *< 0.05, respectively, and = denotes that the results are not significantly different. We also report their Area under curve (AUC), Accuracy (ACC) and MCC for the per-residue disorder predictions generated by the ten considered predictors.

The overall disorder content was used in the past to estimate the abundance of intrinsic disorder in several protein databases [45,46], in various protein families and classes [47-58], and in complete proteomes [59-62]. The high values of the disorder content were reported for several disease-related proteins [2-9]. The content was also used for the analysis of intrinsic disorder-related protein functions [63-65]. Importantly, in all these and similar cases, the disorder content was evaluated based on the results of either binary classifiers or was derived from the per-residue disorder predictions. As mentioned above, these per-residue disorder prediction methods may over-or under-predict the overall amount of disorder in the sequence. This observation and the fact that the knowledge of the disorder content in a given protein or in a set of proteins of interest or in an entire proteome can be utilized to investigate numerous important hypotheses motivate the development of new computational tools for the accurate prediction of the disorder content.

We propose a novel method, named DisCon (Disorder Content predictor), that aims to provide accurate sequence-based predictions of the disorder content. Our approach is based on the premise that sequence-level aggregation of information may provide more accurate content prediction when compared with the content extracted from the local window-based residue-level disorder predictors. DisCon extends the capabilities of the binary predictors from [13,42-44] as it provides a real-value, instead of binary, estimates of the amount of the disorder. Our solution has two key characteristics. *Firstly*, we use a comprehensive selection of the input information sources including sequence, evolutionary profiles generated with PSI-BLAST, and predicted secondary structure, solvent accessibility, B-factors, signal peptides and globular domains. The main reason to use such a diverse set of inputs is to capture different aspects/flavours of disorder [66]. The selection of the first five sources is motivated by their successful use for the residue-level disorder predictions, see Table 2. Similarly as for the ordered proteins, for which the correct folding into biologically active conformations is determined by their amino acid chain, the absence of rigid structure in the intrinsically disordered proteins or regions is also encoded in their amino acid sequences [67,68]. The disordered regions are usually depleted in so-called order-promoting residues (Trp, Tyr, Phe, Ile, Leu, Val, Cys, and Asn) while they include larger numbers of the disorder-promoting residues (Ala, Arg, Gly, Gln, Ser, Glu, Pro, and Lys) [10,42,67,69,70]. Moreover, the disorder is often observed in parts of the sequence that are characterized by low complexity, higher number of Pro and charged residues, and lower amount of hydrophobic and bulky amino acids [10,12,13,27,71], which motivates the use of the input protein chain. The predicted secondary structure is useful since many of the disordered regions are characterized by lack of the secondary structure [14,33,66,69,71]. These unstructured regions usually have a large solvent-accessible area [38] which motivates the application of the predicted solvent accessibility. High values of B-factors are often associated with disordered regions [72], which is why we use their predictions to implement inputs to DisCon. The signal peptides were previously found relevant in the context of analysis of differences between disorder predictors [73] and residues in domains are less likely to be disordered and this information was used in the NORSnet predictor [33]. *Secondly*, we perform a careful design of the input features that are computed based on the aggregation of values for each information source and also by combining information sources. We also designed several customized features that quantify the size and relative location of the predicted secondary structure segments.

**Table 2:** List of input information sources used by the disorder predictors considered in this work.

Prediction method	Method type	Inputs						Data sources for the training/benchmark dataset(s)	Reference
				
		AA sequence	PSI-BLAST	Secondary structure prediction	Solvent accessibility prediction	B-factor prediction	Other		
DISOPRED2	Machine learning	X	X	X				PDB x-ray structures	[22]
IUPred	Relative propensity	X					Energy profile	PDB x-ray structures + curated chains	[17]
PROFbval	Machine learning	X	X	X	X			PDB x-ray structures	[29]
NORSnet	Machine learning	X	X	X	X	X	Predicted protein-protein interfaces, predicted domains	DisProt + PDB x-ray structures	[33]
Ucon	Relative propensity	X					Predicted residue contacts	DisProt + PDB x-ray structures	[19]
DISOclust	Predicted 3D structure	X					3D models	CASP7 + DisProt	[41]
MD	Meta approach	X	X	X	X	X	Predicted disorder	DisProt + PDB x-ray structures	[38]
PONDR-FIT	Meta approach						Predicted disorder	DisProt + PDB x-ray structures	[40]
MFDp	Meta approach	X	X	X	X	X	Predicted disorder	DisProt + PDB x-ray structures	[39]

Methods are sorted in the ascending order by their year of publication.

We empirically demonstrate that the DisCon's predictions are more accurate than the content extracted from the residue-level annotations generated by modern disorder predictors, including methods listed in Table 2. One of the potential applications of the predicted disorder content is to adjust the cut-offs used by the disorder predictors to annotate the disordered residues. We show that these annotations can be improved when the threshold values is adjusted for each chain such that the amount of the predicted disordered amino acids matches not only the native but also the predicted content.

## Methods

### Definition of Disorder

In the past CASP experiments the disordered residues were defined as the amino acids that lack coordinates in their crystal structures and, in the case of the structures solved by NMR, as the amino acids that exhibit high variability within an ensemble or that were annotated by experimentalists as disordered in the REMARK 465 [74,75]. Another commonly used source for the disorder annotations is based on the experimentally-validated and biologically relevant disordered segments from the DisProt database [76]. We note that the assignment of the disordered regions using different experimental methods was previously shown to be potentially inconsistent [66]. Consequently, the disorder predictors that were developed using annotations provided with one method could lead to larger errors when used to predict annotations generated with the help of other methods [19,33]. Therefore, we created a dataset that combines the CASP-defined annotations with the DisProt annotations.

### Datasets

The proposed method was designed and tested using a dataset that was developed to validate a recent meta-predictor of disordered residues, the MFDp [39]. The protein chains were collected from the Protein Data Bank (PDB) [77] and the DisProt [76] databases. The culled PDB list [78] was used to derive a high-quality and low sequence identity subset of the PDB protein. More specifically, only the proteins for which the structure is characterized by R-factor < 0.2 and resolution < 2.0Å, and that are characterized by sequence identity < 25% were kept. We randomly selected 20% of the fully structured proteins among the resulting chains. This is motivated by the fact that many of chains selected using the culled PDB list are annotated as ordered while a recent study shows that completely ordered proteins are not highly abundant in PDB [46]. The PDB chains were combined with all 523 proteins from the release 4.9 of the DisProt. The resulting dataset was filtered to reduce the pairwise sequence identity to below 25% by removing similar sequence with fewer disordered residues. Among the remaining 514 chains we removed four for which MD failed to produce predictions; this also resulted in lack of predictions from Ucon, PROFbval and NORSnet that are bundled with the MD predictions. Moreover, we improved the annotations of the DisProt chains using the procedure described in [79]. We applied the approach based on the SL dataset [79] that combines the disorder annotations from the DisProt with the annotations of disorder and order based on the corresponding structural domains that can be found in PDB. We note that in contrast to the SL dataset that is based on the release 4.5 of DisProt, our annotations are based on the newer release 4.9. Finally, we also removed the HIS-tags that are introduced to ease the crystallization. The resulting dataset includes 305 chains from DisProt and 205 from PDB. This dataset was divided at random into two subsets, the training dataset with 310 chains and the test dataset with the remaining 200 chains.

We note that although there is some overlap between the training and test sequences (depending on the alignment tool used), they are mostly independent at the 25% similarity level. The training dataset was used to develop the predictor including selection of the input features and the parameterization of the prediction model, which were performed based on the 5-fold cross validation protocol. Next, our predictor that was computed using the training dataset was compared with the existing per-residue prediction methods using the test dataset. The training and test datasets are available at http://biomine.ece.ualberta.ca/DisCon/.

### Evaluation criteria

The disorder content predictions are evaluated using three measures:

where *y_i _*∈ *Y *is the native and *x_i_*∈ *X *is predicted disorder content for the *i*^th ^protein chain, *avg_X _*and *avg_Y _*are the sample means of *X *and *Y, s_x _*and *s_y _*are the sample standard deviations of *X *and *Y*.

Following [19,22,33,39] the binary, per-residue disorder predictions and the per-chain predictions of disorder content (protein categorized based on a given disorder content cut-off) were assessed using two measures:

where *TP *is the number of true positives (correctly predicted disordered residues), *FP *denotes false positives (structured residues that were predicted as disordered), *TN *denotes true negatives (correctly predicted structured residues), and *FN *denotes false negatives (disordered residues that were predicted as structured). Accuracy quantifies the overall success rate, i.e., fraction of correct predictions among all prediction, but since it may lead to misleading results when the dataset is unbalanced (which is the case here since majority of residues are structured) we also use MCC. The MCC values range between -1 and 1 and they are equal zero when all residues are predicted to be structured or to be disordered. Higher values of PCC, accuracy and MCC and lower values of MSE and MAE correspond to better predictions. We also evaluated the real-value, per-residue disorder predictions based on the area under the ROC curve (AUC) measure.

### Overview of the proposed predictor

**Figure 1:**
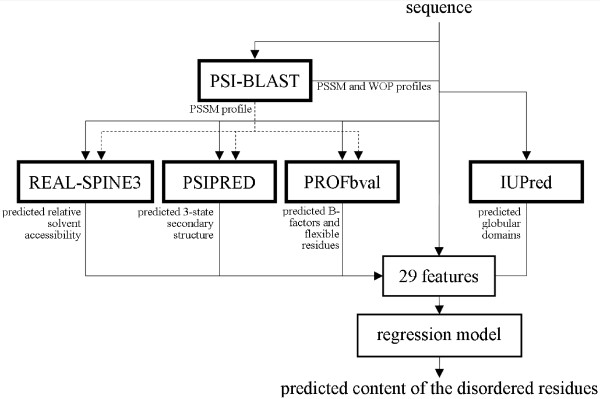
**Architecture of the DisCon predictor**.

The prediction of the disorder content is performed in three steps, see Figure 1. *First*, the input protein chain is processed through PSI-BLAST [80] to generate Position Specific Scoring Matrix (PSSM) and Weighted Observed Percentage (WOP) profiles using the NCBI's nr database downloaded on Nov 19^th ^2009, which was filtered using PFILT [81] to remove low-complexity regions, transmembrane regions, and coiled-coil segments. We use PSIPRED [82] to obtain the 3-state predicted secondary structure (SS), Real-SPINE3 [83] for the prediction of the relative solvent accessibility (RSA), PROFbval [29] for the B-factor and residue flexibility predictions, and IUPred [17] to predict globular domains. The former three methods use the PSSM profiles as their inputs. *Second*, the above predictions, profiles and sequence are used to generate a set of numerical descriptors/features. These features quantify the information encoded in each of the predictions/profiles and also between multiple predictions/profiles. We performed feature selection to select a small subset of 29 features that are relevant to the prediction of the disorder content. *Third*, the selected descriptors are fed into a ridge regression model to generate the predictions.

### Feature-based encoding of the input protein sequence

The input sequence is processed to generate predictions of the 3-state SS, RSA, normalized [72] real-value B-factors, binary annotation of the residue flexibility as provided by PROFbval in two modes, the strict and the non-strict [29], binary annotation of residues that form globular domains, and sequence profiles encoded using PSSM and WOP. We normalized the ASA values predicted by Real-SPINE3 using the maximal ASA values provided in [83] and we preprocessed the 3-state SS by converting the predicted helices that had < 3 residues into coils. We also binarized the real-values RSA to annotate the residues as either solvent exposed when RSA > 0.25 or buried when RSA ≤ 0.25; this cut-off value was used in past studies [72,84]. We also attempted to use signal peptide prediction provided by RPSP [85], but these features were removed during the feature selection. Detailed description of features is provided in Table A1 in the Additional File 1. We generated total of 614 features that are based on

- composition of amino acids

- length and relative location of predicted helix, strand and coil segments

- composition of solvent exposed residues

- composition of flexible residues and sequence segments composed of flexible residues

- number and size of sequence segments that correspond to predicted globular domains

- composition of residue predicted as signal peptides

- fusion of the information coming from multiple predictions, including SS states, solvent exposure, flexibility, and domain annotations. We consider all combinations of two, three and four of the above predictions.

- aggregations of the sequence profiles using entropy and relative (using background probability) entropy by both rows and columns of the PSSM and WOP

- entropy-based aggregations of the sequence profiles encoded with PSSM and WOP which is performed for specific amino acid types, and for residues characterized by specific SS state, solvent exposure, flexibility, and domain annotations.

We emphasize that most of the features, in particular the features that are based on the secondary structure segments, flexible sequence segments, and that combine multiple predicted properties, are novel and unique to this work.

### Design of the predictive model

The features were generated to comprehensively cover information that can be extracted from each predicted property, sequence and sequence profile, and their combinations. Consequently, some of these inputs may not be relevant to the prediction of the disorder content and some could be redundant with each other. We performed two-step feature selection to find a small set of non-redundant and relevant features; the second step also includes computation and parameterization of the predictive model. First, we remove the irrelevant and redundant features using a coarse-grained evaluation based on correlation, and next we perform a wrapper-based selection using the remaining features.

In the *first *step, for each feature we compute its average PCC with the disorder content (the PCC values are based on 5-fold cross validation on the training dataset and they are averages of the coefficients computed in the five training folds) and we remove the features with average absolute PCC value < 0.2. We selected the 0.2 cut-off as this value corresponds to a visible dip in the distribution of the correlation values, see Figure A1 in the Additional File 1. Next, we filtered the remaining 322 features to remove redundancy by assuring that the maximal average absolute PCC value between any pair of these features is < 0.9. Starting with the feature that has the highest average absolute PCC with the native content, we added another feature into the set of filtered features if the average (over the five training folds) absolute PCC between this feature and each feature which is already in the set of filtered features was < 0.9.

In the *second *step, we use the remaining 152 features to perform wrapper-based selection in which a subset of features that results in favourable performance in prediction of the disorder content is retained. We consider two types of predictors, ridge regression and Support Vector Regression (SVR) [86]. The selection of the regression model is motivated by its successful application in several related areas, including evaluation of peptide identification [87] and prediction of folding rates [88,89], solvent accessibility [90], secondary structure content [91], and affinity of protein-ligand complexes [92], to name a few. Similarly, the SVR also enjoys a wide range of relevant applications including prediction of B-factors [93], solvent accessibility [94], and half-sphere exposure [95]. The values of the regression coefficients and the SVR models were estimated from the data in the training folds using WEKA workbench [96]. We consider three types of kernel functions to build SVR models, polynomial, Radial Basis Function (RBF), and Pearson VII function-based Universal Kernel (PUK) [97]. We parameterized each kernel and the complexity constant *C *by performing grid search. We use linear and quadratic polynomials, and *C *equal 2*^x ^*where *x *= -8, -7,..., 2; the RBF kernel with gamma (spread) equal 2*^y ^*where *y *= -11, -10,..., 2, and *C *values where *x *= -3, -2,..., 6; and the PUK kernel with omega equal 2*^z ^*where *z *= -4, -3,..., 1, and *C *values where *x *= -4, -3,..., 5. We also parameterized the ridge parameter in the ridge regression; we considered ridge values equal 10*^w ^*where *w *= -11, -9,..., 2. We first parameterized these 4 predictors (3 SVM types + 1 ridge regression) using a representative subset of the 152 features. We selected one features with the highest average absolute PCC from each of the feature groups defined in Table A1 in the Additional File 1. The representative subset includes 23 features since that number of groups was covered among the 152 features. Next, these parameterized predictors were used to perform feature selection in which we searched for a subset of features that results in the best MSE value. We performed forward and backward best first searches. The forward/backward best first search starts with the empty/entire (152 features) set of features, and it adds/removes one feature at the time if it decreases/increases the MSE value. The search stops when the entire list of features is scanned. As a result, we obtained 8 configurations of 4 predictors with 2 search types. The predictors in each configuration were parameterized using the grid search as described above. The parameterizations and all steps of the feature selection were executed based on multiple repetitions of 5-fold cross validations on the training dataset, and they aimed to minimize the average MSE score between the predicted and the native disorder content. We repeated the cross validations for up to five times using randomized division into the 5 folds for as long as the coefficient of variation (the ratio of the standard deviation to the mean) was below 0.02; this approach should assure a robust estimate of the MSE values. The parameters of the four predictors and the corresponding number of the selected features are given in Table A2 in the Additional File 1. The predictive performance, which was evaluated based on 5-fold cross validation on the training dataset, for the eight configurations is summarized in Table A3 in the Additional File 1. The best performance, in terms of the MSE and PCC values, is achieved with the ridge regression that uses 29 features selected using the forward best first search, and this configuration is used to implement the proposed DisCon predictor.

## Results and Discussion

### Disorder content prediction

We compare the performance of the DisCon with the results obtained using the disorder content computed from the disorder predictions generated by DISOPRED2, IUPred (both versions, IUPredL and IUPredS), PROFbval, NORSnet, Ucon, DISOclust, MD, PONDR-FIT, and MFDp methods. For the per-residue predictors we used the web servers or standalone implementations provided by the authors, and we calculated the content by counting the number of residues predicted as disordered and dividing it by the length of the corresponding protein chain. The results are computed on the test dataset with 200 chains which shares low identity to chains in our training dataset. We note that the methods we compare with use training datasets that may share higher similarity with the chains in our dataset, which could inflate their predictive quality. We also analyze statistical significance of the differences between the content predicted by DisCon and the other methods. We compare the per-chain values of the absolute errors and the squared errors over the 200 chains in the test dataset and the Pearson correlation coefficients computed for 200 randomly selected sets of 100 proteins from the test dataset. Since the measurements follow normal distribution (evaluated using Shapiro-Wilk test at 0.05 significance) we apply the paired t-test and we measure significance of the differences at 0.05 and 0.01 levels. We evaluate the extent of the over-and under-prediction of the disorder content by quantifying the number of the over-and under-predicted chains and the corresponding MAE values and we also provide the AUC, accuracy, and MCC values for the 10 considered per-residue predictors. The results are summarized in Table 1.

The DisCon is shown to provide favourable predictive performance. It obtains MSE equal 0.05 and PCC equal 0.68 on the test dataset. We note that these results are consistent with the results obtained based on the 5-fold cross validation on the training dataset (PCC = 0.70, MSE = 0.05; see Table A3 in the Additional File 1). On the test set, the best performing per-residue disorder predictors are worse than DisCon by 0.016 MSE and 0.07 PCC for the disorder content prediction. The average absolute error of DisCon equals 0.156 when compared with value at or over 0.167 obtained with the current disorder predictors, except for IUPredS for which MAE = 0.155. The improvements in MSE and PCC offered by DisCon are shown to be statistically significant when compared with all considered competitors. The MAE values computed from our predictions are significantly better than the errors based on the predictions with four existing methods and are equivalent with the remaining six predictors. Further analysis reveals that the quality of the DisCon predictions is better for longer chains, while some other methods may produce favorable predictions for short chains. Figure A2 in the Additional File 1, which shows the relation between the chain length and the absolute errors generated by the DisCon and the top-three methods from Table 1, i.e., IUPRedS, Ucon, and PONDR-FIT, demonstrates that the proposed predictor is characterized by smaller absolute errors for longer chains, while the other three predictors on average provide more accurate predictions for short chains. DisCon provides relatively balanced predictions with similar number of over-and under-predicted chains and low MAE values for these two types of errors. We observe that PROFbval and DISOclust are characterized by substantial levels of the over-prediction of the disorder content that are expressed by the large number of the over-predicted chains and/or high MAE for the over-predicted chains. The under-prediction of the disorder content is characteristic for the NORSnet method. Table 1 also shows that the Ucon which obtains relatively low MSE and mid-range PCC is characterized by lower quality of the per-residue predictions with MCC = 0.28.

### Binary prediction of the disorder amount

**Figure 2:**
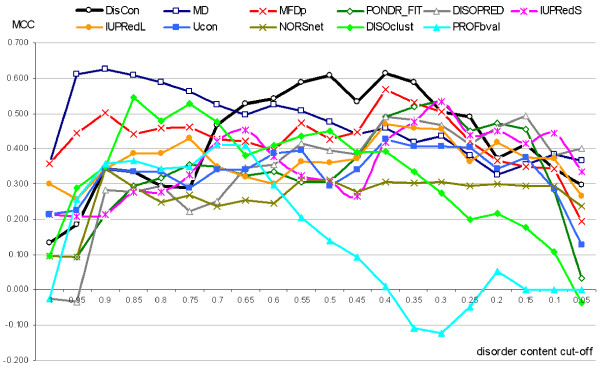
**The MCC values (*y*-axis) for the binary prediction of chains that are characterized by the amount of the disorder below/above a cut-off value shown on the *x*-axis**. The binary predictions are computed by thresholding the predicted disorder content generated by DisCon and the 10 considered disorder predictors on the test dataset.

We apply the predicted disorder content on the test dataset to perform binary prediction of chains that are characterized by the amount of the disorder below/above a specific cut-off value. The cut-offs at 1 and 0 corresponds to detection of fully-disordered and fully-ordered proteins, respectively, while the intermediate cut-off values could be used to find partially structured chains. We measure the MCC of these binary predictions defined using the cut-off values between 0 and 1 with step of 0.05, and the content predicted by DisCon and extracted from the outputs of the 10 considered disorder predictors, see Figure 2. We observe that the usage of the content predicted by DisCon results in the highest MCC values, which range 0.53 and 0.61, for cut-offs between 0.35 and 0.65 inclusive. The best results for the cut-off values above 0.65 are achieved with the content predicted with MD while several predictors do comparably well for the cut-offs below 0.35. We note that the prediction of the fully-structured and fully-disordered proteins could be also accomplished using specialized predictors, such as NN-CDF [44].

### Content guided thresholding of the real-value disorder prediction

**Figure 3:**
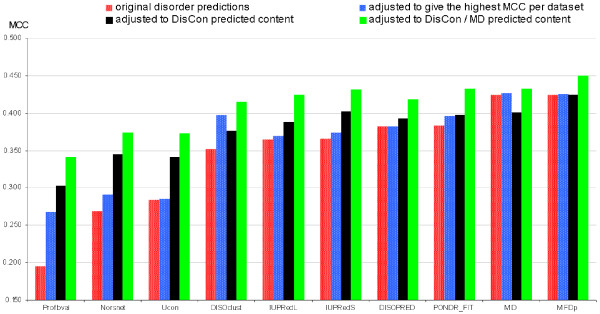
**The MCC values for the residue-level disorder prediction adjusted using content predicted by DisCon**. The bar chart includes the original predictions (densely dotted red bars), predictions with a fixed cut-off that is optimized to maximize MCC on the entire test dataset (sparsely dotted blue bars), predictions where the content predicted by DisCon is used to adjust the cut-off (solid black bars) and where the content predicted by MD if its values are > 0.65 or < 0.1 and otherwise content predicted by DisCon are used to adjust the cut-off (solid green bars). The results were computed on the test dataset and the methods on the *x*-axis are sorted by their original MCC values.

The disorder predictors usually provide both the real-value propensity of the disorder and the binary order/disorder assignment for each residue. The binary assignment is usually based on thresholding of the real-value propensities with a fixed cut-off. We investigate whether the predicted disorder content could be used to guide the selection of the threshold value. This means that instead of using the fixed cut-off we adjust the threshold such that the amount of the residues annotated as disordered equals to the amount of the disorder content predicted with DisCon (using the predictions on the test dataset). We evaluate the binary residue-level disorder prediction of the original and the content-adjusted disorder predictors using MCC, see Figure 3, and accuracy, see Figure A3 in the Additional File 1. We note that the 10 methods that are considered in these figures provide continuous (real-value) prediction values that represent the propensity for a residue to be disordered. We compare the original binary disorder predictions, predictions that are based on a fixed cut-off that maximizes the MCC of a given predictor on our test dataset (to remove a potential bias due to the usage of a different or less complete, i.e., older, disorder annotation to select the original cut-off), and predictions where the cut-off is selected to match the content predicted by DisCon. Based on the Figure 2 that shows that MD outperforms DisCon when the native content < 0.1 or > 0.65, we also consider combining content predicted by the MD and DisCon to adjust the threshold. If the content predicted by MD is > 0.65 or < 0.1 for a given chain then we use the MD predicted content; otherwise we use the content predicted by the DisCon method.

The thresholding of the predicted real-value disorder using the content predicted by DisCon leads to improvements in both MCC and accuracy for all predictors except for the MD, in which case the accuracy is slightly improved but the MCC is lower. The average (across all methods) improvement in MCC and accuracy equal 0.03 and 0.05, respectively. When we use the combination of the content predicted with DisCon and MD the improvements are more substantial and they range between 0.01 and 0.14 for the MCC (on average 0.06) and 0.01 and 0.24 for the accuracy (on average 0.05); the best MCC is obtained using the predictions from the MFDp and it equals 0.45 when compared with 0.425 that was obtained without the content-based adjustment. Interestingly, using this cut-off adjustment the MCC values obtained by seven out of ten considered predictors are > 0.4 while originally (with the default cut-offs), see Table 1, only two methods have MCC > 0.4. This suggests that majority of the considered disorder predictors differentiate between structured and disordered residues based on their real-value propensities in a given chain with relatively similar quality, but only a few of them can accurately scale the range of the real-value propensities between sequences. The content-guided selection of the cut-offs alleviates the prediction bias, i.e., the tendency to under-or over-predict the amount of disorder. The binary predictions of PROFbval, DISOclust, and NORSnet that are originally characterized by relatively low MCC values and a bias towards either over-or under-prediction, see Table 1, are shown to improve by a wide margin when using the disorder content predicted by DisCon or by the combination of DisCon and MD. We observe that the relatively poor performance of the Ucon method does not stem from the prediction bias but rather from its overall problems with the quality of the residue-level annotations, as evidenced by the relatively low AUC and MCC in Table 1 which is in contrast to the sequence-level amount of disorder that is predicted quite accurately by this method.

**Figure 4:**
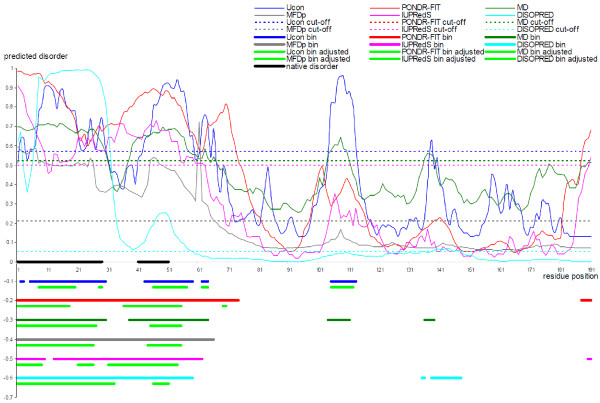
**Prediction of disordered residues in the apoptosis-inducing ligand 2 (Apo2L) protein (PDB ID 1DG6 chain A) by Ucon (thin blue line), PONDR-FIT (thin red line), MD (thin green line), MFDp (thin gray line), IUPredS (thin pink line), and DISOPRED2 (thin cyan line) predictors**. The original cut-offs are shown using dashed lines. The native disordered regions are annotated using black horizontal line. The original binary predictions from Ucon, PONDR-FIT, MD, MFDp, IUPredS, and DISOPRED2 are denoted using blue (at the -0.1 point on the *y*-axis), red (at the -0.2), green (at the -0.3), gray (at the -0.4), pink (at the -0.5), and cyan (at the -0.6) horizontal lines. The binary predictions that were adjusted to match content predicted with DisCon are shown using horizontal bright green lines located immediately under the lines that show the original predictions.

We visualize the improvements that result from the cut-off adjustments using two case studies, one where the original predictions over-estimate the native amount of disorder and another where the predictions are under-estimated. In both cases, we compare the original binary annotations of disordered residues with the annotations that are adjusted using the content predicted with DiscCon, and we include predictions from the top six methods from Table 1, i.e., Ucon, MD, MFDp, PONDR-FIT, IUPredS, and DISOPRED2. The first example is the apoptosis-inducing ligand 2 (Apo2L) protein (PDB ID 1DG6 chain A), see Figure 4. This protein was solved using high-resolution (at 1.3 Å) X-ray crystallography and the structure includes two relatively short disordered segments in the vicinity of the N-terminus [98]. Figure 4 reveals that all six predictors annotate disorder at the N-terminus and that PONDR-FIT and IUPredS also predict a short disordered segment at the C-terminus. However, the disorder at the N-terminus is over-predicted; the residues between positions 30 and 40 and between 52 to about 60 are predicted as disordered, while the X-ray structure shows them as structured. These over-predictions were minimized when the cut-off was adjusted to match the content predicted by the DisCon. Importantly, the adjusted predictions identify the two disordered segments, with particularly good results for the MD and MFDp predictors that quite accurately identify both of the disordered segments and no other disordered residues. After the adjustment, the predictions from PONDR-FIT show the two disordered segments at the N-terminus, although the first segment is predicted to be 11 residues too short, and the C-terminus is predicted as structured. Similarly, the adjusted predictions from IUPredS and DISOPRED2 show disorder in the vicinity of the N-terminus, while the disorder predictions in other parts of this chain are removed.

**Figure 5:**
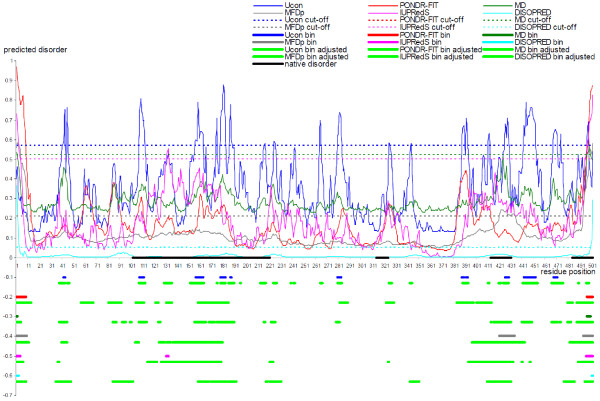
**Prediction of disordered residues in the inosine-5'-monophosphate dehydrogenase protein (DisProt ID DP00399) by Ucon (thin blue line), PONDR-FIT (thin red line), MD (thin green line), MFDp (thin gray line), IUPredS (thin pink line), and DISOPRED2 (thin cyan line) predictors**. The original cut-offs are shown using dashed lines. The native disordered regions are annotated using black horizontal line. The original binary predictions from Ucon, PONDR-FIT, MD, MFDp, IUPredS, and DISOPRED2 are denoted using blue (at the -0.1 point on the *y*-axis), red (at the -0.2), green (at the -0.3), gray (at the -0.4), pink (at the -0.5), and cyan (at the -0.6) horizontal lines. The binary predictions that were adjusted to match content predicted with DisCon are shown using horizontal bright green lines located immediately under the lines that show the original predictions.

The second case is the inosine-5'-monophosphate dehydrogenase (DisProt ID DP00399) which was also solved using X-ray crystallography, see Figure 5. This protein includes four disordered segments, one longer between positions 102 and 221, and three shorter towards the C-terminus [99]. Overall, the six predictors under-predicted the disorder levels in this protein. They predicted only a few disordered residues at both termini, with the exception of Ucon that predicted about a dozen of short disordered segments throughout the entire chain and MFDp that predicted three disordered segments, including both termini and a segment between positions 421 and 434. The DisCon predicted 28.6% of residues to be disordered, when compared with 1%, 1.2%, 2.8%, 3%, 6.6%, and 12% that were predicted by the DISOPRED2, MD, IUPredS, PONDR-FIT, MFDp, and Ucon, respectively; the native amount of disorder is 33.7%. The content-adjusted annotation of the disordered residues captures a large number of disordered residues in the long segment between positions 102 and 221, as well as the two disordered segments nearest to the C-terminus. These improvements come as a trade-off for an over-prediction of the disorder at the N-terminus, particularly for the PONDR-FIT and DISOPRED2 predictors. Overall, we observe that the adjusted predictions show a denser concentration of the disordered residues around the natively disordered regions.

We conclude that although predictions shown in the two case studies should not be assumed typical, they demonstrate that the content predicted with DisCon offers valuable assistance in selection of the cut-offs to annotate the disordered residues based on the real-values predictions from modern disorder predictors.

### Factors related to the amount of disorder/order

**Figure 6:**
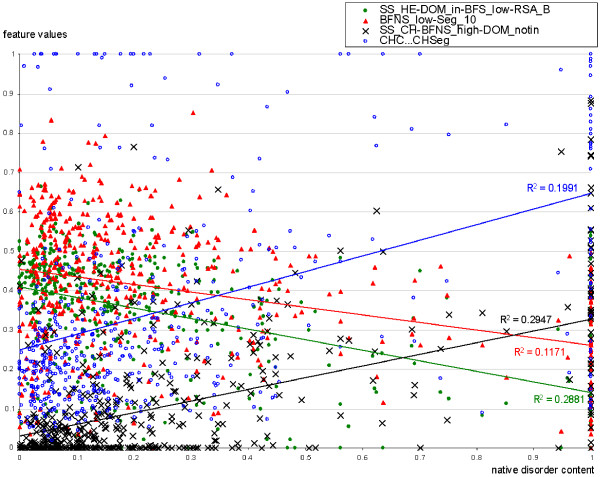
**Scatter plots of the relations between the values of selected four input features, SS_HE-DOM_in-BFNS_low-RSA_B (green circle markers), BFNS_low-Seg_10 (red triangle markers), SS_CH-BFNS_high-DOM_notin (black × markers), and CHC...CHSeg (blue hollow circle markers) shown on *y*-axi**s **and the native disorder content given on *x*-axis**. The lines correspond to linear regressions with the corresponding R^2 ^values.

We convert the input protein sequence into a custom-designed set of selected 29 numerical descriptors which utilize information related to the evolutionary profiles, sequence itself, and predicted secondary structure (SS), solvent accessible residues (RSA), B-factors and globular domains; see Table A4 in the Additional File 1. Majority of the selected features combine multiple input sources. For instance, the largest group of 5 similar features is based on counting the residues in certain predicted SS states, with certain levels of predicted solvent exposure and B-factors which are located within the predicted domains. For instance, the SS_HE_DOM_in_BFNS_low_RSA_B _feature counts the predicted helix and strand residues that are in globular domains, have low B-factors (are structurally rigid) and are buried. As expected, these values are negatively correlated with the content of the native disorder (with PCC = -0.54 in our dataset), see Figure 6, since this descriptor highlights hallmarks of well-structured proteins, i.e., they usually include conserved domains with buried strands and/or helices that are usually structurally rigid. Another feature that attains negative, -0.34, correlation with the native content is BFNS_low_Seg_10_, which quantifies the number of predicted rigid residues (with low B-factor) that are grouped together in the sequence in segments of size at least 10. Figure 6 shows a cluster of BFNS_low_Seg_10 _values between 0.4 and 0.7 for chains with low amount of native disorder, which suggests that well-structured proteins include significant amount of rigid residues that are grouped together in the sequence, while disordered chains contain fewer of such rigid residues. We also discuss two features that have relatively high positive correlation with the disorder content. The SS_CH_BFNS_high_DOM_notin _feature counts the number of coil and helix residues with high B-factor that are not in the globular domains. This feature that has PCC = 0.54 in our dataset, see Figure 6, agrees with characteristic properties of the disorder, which often concerns flexible residues that are outside of the domains. The CHC...CHSeg feature (PCC = 0.45 in our dataset) computes the number of residues in the longest segment in the predicted SS that does not include strands, i.e., stretch of the sequence that is composed only of a coils or of alternating helix-coil-helix-coil... segments. This feature demonstrates that disordered proteins are often depleted of beta-sheets.

The input features also highlight the importance of the relation between sequence conservation and the amount of the disorder, i.e., 13 out of the 29 features utilize entropy computed from the PSSM or WOP profiles. For instance, the EntAvePSSM feature, which computes the entropy of the average PSSM scores for each column (amino acid type) in the matrix along the sequence, has PCC = -0.5. This means that well-structured proteins are characterized on average by a stronger degree of sequence conservation when compared with the disordered proteins. Our observation is in agreement with the results of previous study, where the evolution rates of ordered and intrinsically disordered regions were compared using the pairwise genetic distances between the ordered and the disordered regions of 26 protein families having at least one member with a structurally characterized region of disorder of 30 or more consecutive residues [100]. This study demonstrated that the disordered regions evolved significantly more rapidly than the ordered regions in 19 of the 26 families studied [100].

## Conclusions

In spite of the fact that the quality of the high-throughput disorder prediction continues to improve [75], researchers recognize that new and more accurate predictors are still needed [38,39]. We address the shortage of accurate methods that predict the overall amount of disorder in a given protein chain, which is motivated by the fact that current disorder predictors tend to provide relatively inaccurate estimates of the disorder content. We propose a novel approach, called DisCon, which combines information derived from sequence, sequence profiles, and predicted secondary structure, solvent accessibility, flexibility, and annotation of globular domains. We custom designed feature-based representation of the input protein chain that aggregates and combines these inputs and we performed feature selection that found a small set of 29 complementary features that are well correlated with the native disorder. Using these features and a ridge regression-based model, the DisCon predicts the disorder content with low, 0.05, mean squared error and high, 0.68, correlation, as evaluated on an independent test dataset. These predictions are empirically shown to be significantly better than the disorder content estimates derived from outputs of ten modern disorder predictors. The DisCon's predictions provide a high-quality alternative for high-throughput annotation of the disorder content. They are also shown to provide useful input to improve binary annotations of the disordered residues from the real-value disorder propensities generated by current disorder prediction methods.

## List of abbreviations

AUC: Area Under the ROC Curve; ASA: Absolute Solvent Accessibility; CASP: Critical Assessment of Techniques for Protein Structure Prediction; CDF: Cumulative Distribution Function; CH-plot: Charge-Hydropathy plot; DisCon: Disorder Content predictor; IDP: Intrinsically Disordered Protein; MAE: Mean Absolute Error; MCC: Matthews Correlation Coefficient; MSE: Mean Squared Error; NMR: Nuclear Magnetic Resonance; PCC: Pearson Correlation Coefficient; PDB: Protein Data Bank; PSSM: Position Specific Scoring Matrix; PUK: Pearson VII function-based Universal Kernel; RBF: Radial Basis Function; RSA: Relative Solvent Accessibility; SS: Secondary Structure; SVR: Support Vector Regression; WOP: Weighted Observed Percentage.

## Competing interests

The authors declare that they have no competing interests.

## Authors' contributions

MJM contributed to the collection of the data, design of the prediction method, experimental validation, analysis and interpretation of the results, and implementation of the web server. TZ worked on the design and implementation of the prediction method and analysis and interpretation of the results. BX helped with the analysis and interpretation of the results. YZ and AKD helped with the conception of the project and analysis and interpretation of the results. VNU contributed to the analysis and interpretation of the results and writing of the manuscript. LK was responsible for the conception of the project, and contributed to the collection of the data, design and implementation of the prediction method, experimental validation, analysis and interpretation of the results, and writing of the manuscript. All authors have read, corrected and approved the manuscript.

## Supplementary Material

Additional file 1 **Supplementary tables and figures**. This file includes 4 supplementary tables and 3 supplementary figures. The tables summarize the input features and results obtained with alternative designs of the proposed predictor. The figures summarize correlation between the input features and the predictive target, the relation between the predictive quality and the input chain length, and the accuracy for the residue-level disorder predictions. Click here for file
