# Supplementary material for: In-silico prediction of disorder content using hybrid sequence representation
Source: BMC Bioinformatics. 2011 Jun 17;12:245. doi: 10.1186/1471-2105-12-245 (PMC3212983; doi:10.1186/1471-2105-12-245)
Supplement: Additional file 1 — Supplementary tables and figures. This file includes 4 supplementary tables and 3 supplementary figures. The tables summarize the input features and results obtained with alternative designs of the proposed predictor. The figures summarize correlation between the input features and the predictive target, the relation between the predictive quality and the input chain length, and the accuracy for the residue-level disorder predictions. [file 1471-2105-12-245-S1.PDF]

Supplementary materials for "in-silico prediction of disorder content using hybrid sequence representation"

by Marcin J. Mizianty, Tuo Zhang, Bin Xue, Yaoqi Zhou, A. Keith Dunker, Vladimir N. Uversky, and Lukasz Kurgan

Table A1. Summary of the set of 614 considered features including the inputs data sources that were used to compute them, their names, and descriptions. The last two columns describe the number of features and their grouping, which is based on the input sources that they utilize.

Input sources								Features			
AA[a]	SS[b]	RSA[c]	BF[d]	DOM[e]	SP[f]	PSSM[g]	WOP[h]	Name	Description	Count	Group
								N	Protein chain length	1	1
×								AA_qContent	Number of AA residues divided by N	20	1
	×							SS_iSeg	Length of the longest SS segment divided by N	3	2
	×							TriSS_iSeg	Length of the longest triple SS segment (including the longest SS segment, i.e. 'CHC'-'C'; 'CEC'-'C'; 'HCH'-'H'; 'ECE'-'E') divided by N	6	2
	×							TranSS_k	Number of SS transitions divided by N	3	2
	×							EH-SegCollocated	Number of strand segments next to helix segments (separated by a coil) divided by N	1	2
	×							TranCH	Largest number of coil-helix transitions in a sequence without an intermediate strand segments divided by N	1	2
	×							TranCE	Largest number of coil-strand transitions in a sequence without an intermediate helix segments divided by N	1	2
	×							CHC…CHSeg	Largest number of residues in coil-helix-coil-helix… segments (including the longest coil segment) without an intermediate strand segments divided by N	1	2
	×							CEC…CESeg	Largest number of residues in coil-strand-coil-strand… segments (including the longest coil segment) without an intermediate helix segments divided by N	1	2
	×							SS_iSeg_v	Number of residues in SS segments of length >= v divided by N	9	2
	×							SS_i	Number of residues in SS segments divided by N	3	2
		×						RSA	Number of residues that are solvent exposed divided by N	1	3
			×					AveRealBF	Average value of the B-factors predicted (sum divided by N)	1	4
			×					BFNS	Number of residues that have high B-factor in the non-strict mode	1	4
			×					BFS	Number of residues that have high B-factor in the strict mode	1	4
			×					$BFNS_nSeg_w$	Number of residues that have high (or low) B-factor in the non-strict mode in segments of length >= w divided by N	8	4
			×					BFS_nSeg_w	Number of residues that have high (or low) B-factor in the strict mode in segments of length >= w divided by N	8	4
			×					$BFNS_n$Seg	Number of segments of residues that have high (or low) B-factor in the non-strict mode divided by N	2	4
			×					BFS_nSeg	Number of segments of residues that have high (or low) B-factor in the strict mode divided by N	2	4
			×					$RealBF_z$	Number of residues that have the real-value B-factor > z divided by N	3	4
				×				DOM	Number of residues in domains divided by N	1	5
				×				DOMSeg	Number of domains divided by N	1	5
				×				DOM_mSeg	Length of the longest domain (or non-domain) segment divided by N	2	5
					×			SP	Number of residues predicted as signal peptides divided by N	1	6
	×	×						SS_l-RSA_p	Number of SS residues that are solvent exposed (or buried) divided by N	12	7
	×		×					SS_l-$BFNS_n$	Number of SS residues with high (or low) B-factor in non-strict mode divided by N	12	8
	×		×					SS_l-BFS_n	Number of SS residues with high (or low) B-factor in strict mode divided by N	12	8
	×			×				SS_l-DOM_m	Number of SS residues which are (or not) in domains divided by N	12	9
		×	×					$BFNS_n$-RSA_p	Number of residues that are exposed (or buried) and that have high (or low) B-factor in the non-strict mode divided by N	4	10
		×	×					BFS_n-RSA_p	Number of residues that are exposed (or buried) and that have high (or low) B-factor in the strict mode divided by N	4	10
		×		×				RSA_p-DOM_m	Number of exposed (or buried) residues that are (or not) in domains divided by N	4	11
			×	×				DOM_m-$BFNS_n$	Number of residues that are in (or not in) domains and that have high (or low) B-factor in the non-strict mode divided by N	4	12
			×	×				DOM_m-BFS_n	Number of residues that are in (or not in) domains and that have high (or low) B-factor in the strict mode divided by N	4	12
	×	×	×					SS_l-$BFNS_n$-RSA_p	Number of SS residues with high (or low) B-factor in the non-strict mode that are solvent exposed (or buried) divided by N	24	13
	×	×	×					SS_l-BFS_n-RSA_p	Number of SS residues with high (or low) B-factor in the strict mode that are solvent exposed (or buried) divided by N	24	13
	×	×		×				SS_l-DOM_m-RSA_p	Number of SS residues that are solvent exposed (or buried) and which are (or not) in the domains divided by N	24	14
	×		×	×				SS_l-$BFNS_n$-DOM_m	Number of SS residues with high (or low) B-factor in the non-strict mode and that are (or not) in domains divided by N	24	15
	×		×	×				SS_l-BFS_n-DOM_m	Number of SS residues with high (or low) B-factor in the strict mode and that are (or not) in domains divided by N	24	15

		×	×	×				RSA_p-DOM_m-$BFNS_n$	Number of exposed (or buried) residues that are (or not) in domains and that have high (or low) B-factor in the non-strict mode divided by N	8	16
		×	×	×				RSA_p-DOM_m-BFS_n	Number of exposed (or buried) residues that are (or not) in domains and that have high (or low) B-factor in the strict mode divided by N	8	16
	×	×	×	×				SS_l-DOM_m-$BFNS_n$-RSA_p	Number of SS residues that are exposed (or buried), that have high (or low) B-factor in the non-strict mode and that are (or not) in the domains divided by N	48	17
	×	×	×	×				SS_l-DOM_m-BFS_n-RSA_p	Number of SS residues that are exposed (or buried), that have high (or low) B-factor in the strict mode and that are in (or not in) the domains divided by N	48	17
						×		$EntPSSM_r$Content	Number of residues that have the entropy of PSSM > r, divided by N	4	18
						×		$REntPSSM_s$Content	Number of residues that have the relative entropy of PSSM > s, divided by N	4	18
							×	$EntWOP_r$Content	Number of residues that have the entropy of WOP > r, divided by N	4	19
							×	$REntWOP_s$Content	Number of residues that have the relative entropy of WOP > s, divided by N	4	19
						×		$EntPSSM_t$Seg	Length of the longest segment in which residues have the entropy of PSSM in certain range t, divided by N	8	18
						×		$REntPSSM_u$Seg	Length of the longest segment in which residues have the relative entropy of PSSM in certain range u, divided by N	8	18
							×	$EntWOP_t$Seg	Length of the longest segment in which residues have the entropy of WOP in certain range t, divided by N	8	19
							×	$REntWOP_u$Seg	Length of the longest segment in which residues have the relative entropy of WOP in certain range u, divided by N	8	19
						×		AveEntPSSM	Average of the entropy of PSSM per residue	1	18
						×		AveREntPSSM	Average of the relative entropy of PSSM per residue	1	18
							×	AveEntWOP	Average of the entropy of WOP per residue	1	19
							×	AveREntWOP	Average of the relative entropy of WOP per residue	1	19
						×		EntAvePSSM	Entropy of averaged PSSM per each amino acid type along the sequence	1	18
						×		REntAvePSSM	Relative entropy of averaged PSSM per each amino acid type along the sequence	1	18
							×	EntAveWOP	Entropy of averaged WOP per each amino acid type along the sequence	1	19
							×	REntAveWOP	Relative entropy of averaged WOP per each amino acid type along the sequence	1	19
×						×		$EntPSSM_q$	Entropy of PSSM along the sequence for 20 amino acid types	20	20
×							×	$EntWOP_q$	Entropy of WOP along the sequence for 20 amino acid types	20	21
×						×		AveEntPSSM-AA_q	Average of the entropy of PSSM for 20 amino acid types	20	20
×						×		AveREntPSSM-AA_q	Average of the relative entropy of PSSM for 20 amino acid types	20	20
×							×	AveEntWOP-AA_q	Average of the entropy of WOP for 20 amino acid types	20	21
×							×	AveREntWOP-AA_q	Average of the relative entropy of WOP for 20 amino acid types	20	21
	×					×		AveEntPSSM-SS_l	Average of the entropy of PSSM for residues in SS_l segment	6	22
	×					×		AveREntPSSM-SS_l	Average of the relative entropy of PSSM for residues in SS_l segment	6	22
	×						×	AveEntWOP-SS_l	Average of the entropy of WOP for residues in SS_l segment	6	23
	×						×	AveREntWOP-SS_l	Average of the relative entropy of WOP for residues in SS_l segment	6	23
		×				×		AveEntPSSM-RSA_p	Average of the entropy of PSSM for exposed (or buried) residues	2	24
		×				×		AveREntPSSM-RSA_p	Average of the relative entropy of PSSM for exposed (or buried) residues	2	24
		×					×	AveEntWOP-RSA_p	Average of the entropy of WOP for exposed (or buried) residues	2	25
		×					×	AveREntWOP-RSA_p	Average of the relative entropy of WOP for exposed (or buried) residues	2	25
			×			×		AveEntPSSM-$BFNS_n$	Average of the entropy of PSSM for residues that have high (or low) B-factors in the non-strict mode	2	26
			×			×		AveREntPSSM-$BFNS_n$	Average of the relative entropy of PSSM for residues that have high (or low) B-factors in the non-strict mode	2	26
			×				×	AveEntWOP-$BFNS_n$	Average of the entropy of WOP for residues that have high (or low) B-factors in the non-strict mode	2	27
			×				×	AveREntWOP-$BFNS_n$	Average of the relative entropy of WOP for residues that have high (or low) B-factors in the non-strict mode	2	27
			×			×		AveEntPSSM-BFS_n	Average of the entropy of PSSM for residues that have high (or low) B-factors in the strict mode	2	28
			×			×		AveREntPSSM-BFS_n	Average of the relative entropy of PSSM for residues that have high (or low) B-factors in the strict mode	2	28
			×				×	AveEnWOP-BFS_n	Average of the entropy of WOP for residues that have high (or low) B-factors in the strict mode	2	29
			×				×	AveREntWOP-BFS_n	Average of the relative entropy of WOP for residues that have high (or low) B-factors in the strict mode	2	29
				×		×		AveEntPSSM-DOM_m	Average of the entropy of PSSM for residues that are (or not) in domains	2	30
				×		×		AveREntPSSM-DOM_m	Average of the relative entropy of PSSM for residues that are (or not) in domains	2	30
				×			×	AveEntWOP-DOM_m	Average of the entropy of WOP for residues that are (or not) in domains	2	31
				×			×	AveREntWOP-DOM_m	Average of the relative entropy of WOP for residues that are (or not) in domains	2	31

Table legend

[a] Amino acid type; [b] 3-state secondary structure predicted by PSI-PRED; [c] Relative solvent accessibility predicted by RealSPINE3 (residues are categorized as buried if their RSA ≤ 0.25; otherwise they are assumed to be solvent exposed); [d] B-factors (normalized) predicted by PROFbval in strict/non-strict mode; [e] domains predicted by IUpred; [f] signal peptides predicted by RPSP; [g] Position specific scoring matrix generated by PSI-BLAST; [h] Weighted observed percentage generated by PSI-BLAST; i∈{C(coil), H(helix), S(strand)}; j∈{CHC, CEC, HCH, ECE, HCE, ECH}; k∈{CH, CE, CH & CE & HE}; l∈{C, H, E, C&H, C&E, H&E}; m∈{in domain, not in domain}; n∈{high B-factor, low B-factor}; p∈{exposed (RSA>0.25), buried (RSA <=0.25)}; q∈{20 amino acid types}; r∈{0.6, 0.7, 0.8, 0.9}; s∈{1, 2, 3, 4}; t∈{<= 0.6, > 0.6, <= 0.7, > 0.7, <= 0.8, > 0.8, <= 0.9, > 0.9}; u∈{<= 1, > 1, <= 2, > 2, <= 3, > 3, <= 4, > 4}; v∈{10, 15, 20}; w∈{3, 5 , 10, 15}; z∈{0.5, 1, 2}.

Table A2. Number of features and parameters of predictors selected during the design process. All results are based on the multiple repetitions of 5-fold cross validation on the training dataset.

Feature set	Results of parameterizations										
	Ridge regression		SVR polynomial kernel			SVR RBF kernel			SVR PUK kernel		
	# features	Ridge	# features	*C*	exp	# features	*C*	γ	# features	*C*	ω
23 representative features selected from 152 features after the first feature selection step	23	10.0	23	2^{-5}	1	23	8	2^{-9}	23	1	0.25
Features selected using forward best first search	29	10.0	29	2^{-8}	2	33	1	0.5	31	1	0.5
Features selected using backward best first search	37	1.0	31	2^{-4}	1	31	32	2^{-10}	18	4	0.25

Table A3. Summary of the predictive quality obtained with the considered 8 configurations of 4 predictors with 2 search types. The results are based on the 5-fold cross validation on the training dataset and the best values are shown in bold font.

Predictors	Feature selection		Prediction quality		
	search method	# selected features	MSE	MAE	PCC
SVR with RBF kernel	forward best first	33	0.0505	0.1484	0.6741
SVR with RBF kernel	backward best first	31	0.0492	0.1403	0.6846
SVR with PUK kernel	forward best first	31	0.0481	0.1565	0.6891
SVR with PUK kernel	backward best first	18	0.0476	0.1572	0.6931
SVR with polynomial kernel	forward best first	29	0.0501	0.1409	0.6806
SVR with polynomial kernel	backward best first	31	0.0495	**0.1402**	0.6820
Ridge Regression	forward best first	29	**0.0456**	0.1527	**0.7039**
Ridge Regression	backward best first	37	0.0462	0.1516	0.7009

Table A4. Summary of the selected set of 29 features including the inputs data sources that were used to compute them, their names, descriptions and rank (based on their average absolute PCC with the native content in the training dataset) in the feature set. Features are sorted by their ranks and grouped based on the input sources they utilize.

Input sources							Features	
AA[a]	SS[b]	RSA[c]	BF[d]	DOM[e]	PSSM[f]	WOP[g]	Name [rank][h]	Description[i]
	×		×	×			SS_{CH}-$BFNS_{high}$-DOM_{notin} [1]	Number of coil and helix residues with high B-factor in the non-strict mode and that are not in domains
	×						$TriSS_{CHC}$-Seg [2]	Length of the longest coil-helix-coil segment
							$TriSS_{HCH}$-Seg [29]	Length of the longest helix-coil-helix segment
	×	×	×	×			SS_{HE}-DOM_{in}-BFS_{low}-RSA_B [3]	Number of helix and strand residues that are buried, have low B-factor in the strict mode and that are in the domains
							SS_{CH}-DOM_{in}-$BFNS_{low}$-RSA_B [12]	Number of coil and helix residues are buried, that have low B-factor in the non-strict mode and that are in the domains
							SS_C-DOM_{in}-$BFNS_{high}$-RSA_B [13]	Number of coil residues that are buried, have high B-factor in the non-strict mode and that are in the domains
							SS_{CH}-DOM_{in}-BFS_{low}-RSA_E [14]	Number of coil and helix residues that are exposed, have low B-factor in the strict mode and that are in the domains
							SS_{HE}-DOM_{in}-BFS_{low}-RSA_E [17]	Number of helix and strand residues that are exposed, have low B-factor in the strict mode and that are in the domains
					×		EntAvePSSM [4]	Entropy of averaged PSSM per each amino acid type along the sequence
	×						CHC...CHSeg [5]	Largest number of residues in coil-helix-coil-helix… segments (including the longest coil segment) without an intermediate strand segment
					×		$REntPSSM_{<=2}$-Seg [6]	Length of the longest segment in which residues have the relative entropy of PSSM ≤ 2
	×	×		×			SS_C-DOM_{in}-RSA_B [7]	Number of coil residues that are buried and are in the domain
							SS_{CE}-DOM_{in}-RSA_B [8]	Number of coil and strand residues that are buried and are in the domain
				×		×	AveEntWOP-DOM_{in} [9]	Average of the entropy of WOP for residues that are in domains
	×		×				SS_{HE}-$BFNS_{low}$ [10]	Number of helix and strand residues with low B-factor in non-strict mode
			×				BFS_{high}-Seg [11]	Number of segments of residues that have high B-factor in the strict mode
							$BFNS_{high}$-Seg [16]	Number of segments of residues that have high B-factor in the non-strict mode
			×				$BFNS_{low}$-Seg_{10} [15]	Number of residues that have low B-factor in the non-strict mode in segments of length ≥ 10
					×		$EntPSSM_Q$ [18]	Entropy of PSSM along the sequence for Gln (Q)
						×	$EntWOP_W$ [19]	Entropy of WOP along the sequence for Trp (W)
×					×		AveREntPSSM-AA_L [20]	Average of the relative entropy of PSSM for Leu (L)
							AveREntPSSM-AA_S [23]	Average of the relative entropy of PSSM for Ser (S)
					×		$REntPSSM_2$-Content [21]	Number of residues that have the relative entropy of PSSM > 2
							$REntPSSM_3$-Content [27]	Number of residues that have the relative entropy of PSSM > 3
	×				×		AveREntPSSM-SS_{HE} [22]	Average of the relative entropy of PSSM for residues in helix and strand segments
	×					×	AveEntWOP-SS_H [24]	Average of the entropy of WOP for residues in helix segments
×					×		AveEntPSSM-AA_M [25]	Average of the entropy of PSSM for Met (M)
							AveEntPSSM-AA_A [28]	Average of the entropy of PSSM for Ala (A)
×							AA_F-Content [26]	Composition of Phe (F)

Table legend
[a] Amino acid type; [b] 3-state secondary structure predicted by PSI-PRED; [c] Relative solvent accessibility predicted by RealSPINE3 (residues are categorized as buried if their RSA ≤ 0.25; otherwise they are assumed to be solvent exposed); [d] B-factors (normalized) predicted by PROFbval in strict/non-strict mode; [e] globular domains predicted by IUpred; [f] Position specific scoring matrix generated by PSI-BLAST; [g] Weighted observed percentage generated by PSI-BLAST; [h] The features are ranked according to their average (over the 5 cross-validation folds in the training dataset) PCC values against the target (disorder content). Similar features were grouped into the same subsets, which were ranked with respect to the highest rank of all features in that subset; [i] features that count the number of residues or segments were normalized by dividing by N (length of the sequence).

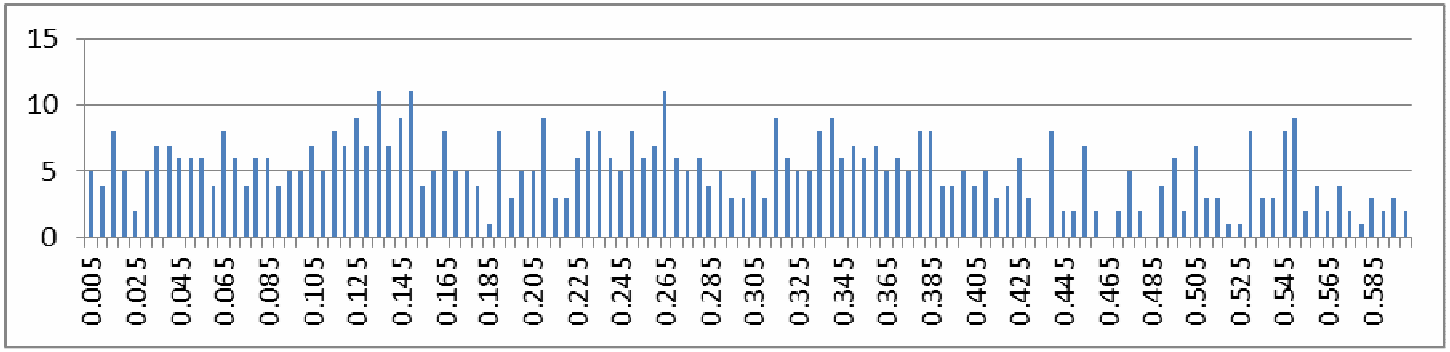

Figure A1. Distribution of the average (over the five training folds in the training dataset) absolute PCC values (shown on the *x*-axis) computed between the values of each of the 614 considered features and the native disorder content. The PCC values are binned into 0.005 wide intervals.

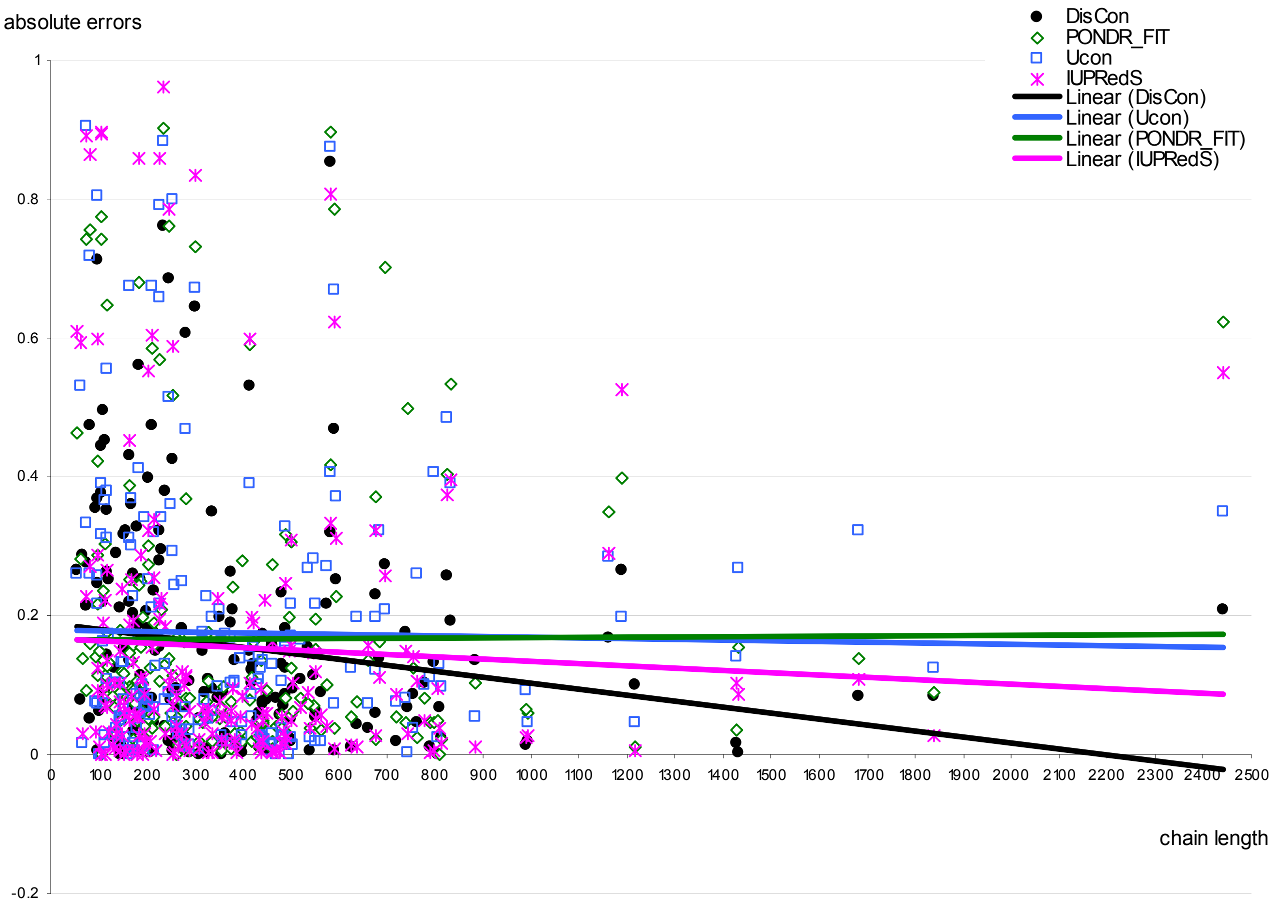

Figure A2. Relation between the mean error of the disorder content prediction (shown on the *y*-axis) and the length of the corresponding chains (shown on the *x*-axis) on the test dataset for the DisCon (solid black circle markers), PONDR-FIT (hollow green diamond markers), Ucon (hollow blue square markers), and IUPredS (pink star markers). The solid lines show linear regressions of the relations between the errors and chain lengths.

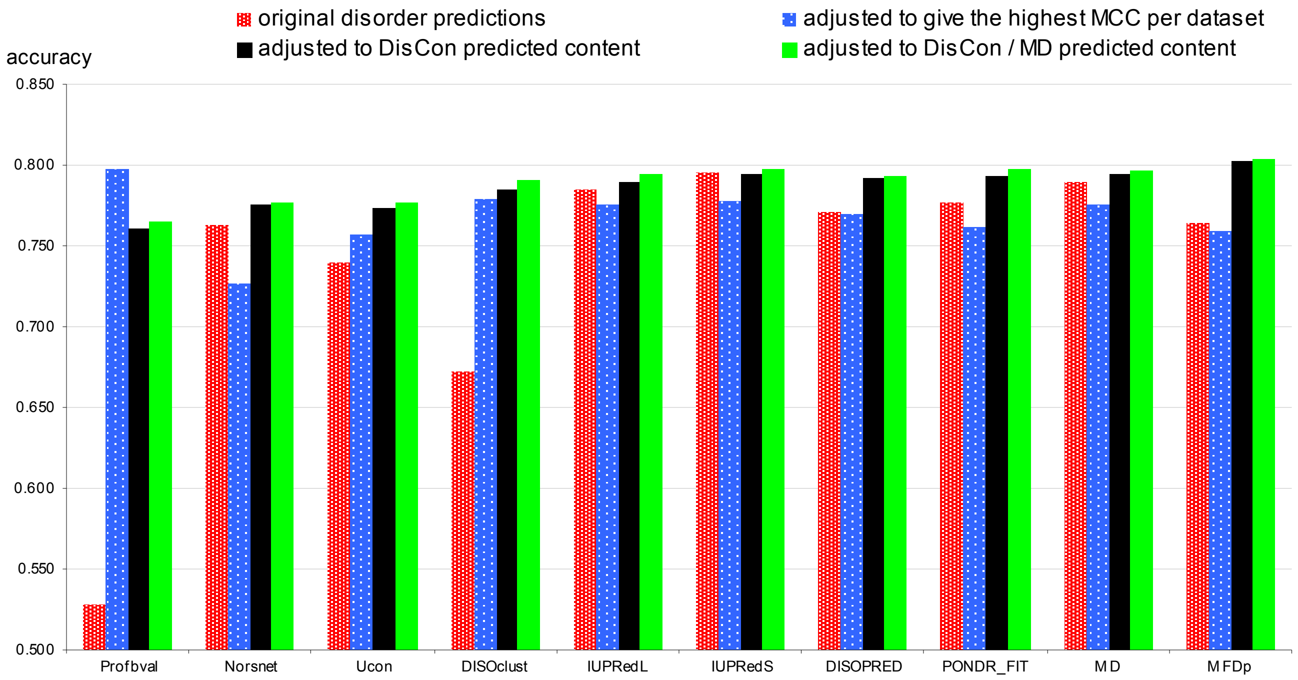

Figure A3. The accuracy values for the residue-level disorder prediction adjusted using content predicted by DisCon. The bar chart includes the original predictions (densely dotted red bars), predictions with a fixed cut-off that is optimized to maximize MCC on the entire test dataset (sparsely dotted blue bars), predictions where the content predicted by DisCon is used to adjust the cut-off (solid black bars) and where the content predicted by MD if its values are >0.65 or <0.1 and otherwise content predicted by DisCon are used to adjust the cut-off (solid green bars). The results were computed on the test dataset and the methods on the x-axis are sorted by their original MCC values (see Table 2 in the manuscript).
